# “*The Simplest Way to Go*”: An Exploration of Why Women Who Inject Drugs Chose Long-Acting Injectable Cabotegravir Instead of Daily Oral PrEP

**DOI:** 10.1007/s10461-025-04833-5

**Published:** 2025-08-06

**Authors:** Alexis M. Roth, Kathleen M. Ward, Erin McDowell, Elana Forman, K. Rivet Amico, Tyler S. Bartholomew, Douglas Krakower, Daniel Vader, Silvana Mazzella, Adam W. Carrico, Susan G. Sherman, Allison Groves

**Affiliations:** 1 Department of Community Health and Prevention, Dornsife School of Public Health, Drexel University, 3215 Market Street, Philadelphia, PA 19104 USA; 2 Department of Health Behavior and Health Education, University of Michigan School of Public Health, Ann Arbor, MI USA; 3 Department of Public Health Sciences, University of Miami Miller School of Medicine, Miami, FL USA; 4 Department of Population Medicine, Harvard Medical School, Boston, MA USA; 5 Division of Infectious Diseases, Beth Israel Deaconess Medical Center, Boston, MA USA; 6 Department of Biostatistics, Dornsife School of Public Health, Drexel University, Philadelphia, PA USA; 7 Prevention Point Philadelphia, Philadelphia, PA USA; 8 Department of Health Promotion and Disease Prevention, Robert Stempel College of Public Health and Social Work, Florida International University, Miami, FL USA; 9 Department of Health, Behavior, and Society, Johns Hopkins Bloomberg School of Public Health, Baltimore, MD USA

**Keywords:** Women, Injection drug use, PrEP, Cabotegravir, Preferences

## Abstract

Long-acting injectable PrEP was approved for use in the US in 2021 but roll out has been slow, with few studies exploring uptake among cisgender women who inject drugs (WWID). We purposively recruited 25 WWID within 30-days of receiving a PrEP prescription from a low-barrier clinic co-located with a syringe services program to complete semi-structured interviews about PrEP product choice. We used an intersectional lens to compare decision-making between women choosing injectable PrEP versus oral PrEP and continued enrolling new participants until we reached thematic saturation (12/2022 to 2/2024). Participants represent a diverse sample of WWID (12 women of color) with median age 43 years. Daily injection drug use (72%) and ≥ 1 sex partner (64%) were common. Salient themes from interviews include: (1) PrEP provides women with a valued safety net and initiation is a form of self-preservation. (2) Co-located care, small incentives, and provider respect for WWID's medical autonomy helped participants navigate a multi-visit PrEP intake process. (3) Longer lasting HIV protection with less frequent dosing is preferrable to a shorter acting daily oral medication. When selecting a product, WWID evaluated its attributes against their medical history and personal circumstances like homelessness (64%). Most chose CAB-LA (72%) because it provides longer lasting protection which was a highly valued product attribute. Together, our findings underscore the critical importance of offering multiple PrEP options when implementing HIV prevention strategies that are patient-centered and responsive to the unique needs of WWID.

## Introduction

Cisgender women who inject drugs (WWID), particularly those engaged in transactional sex, bear a disproportionate burden of HIV [1–3]. Approximately 30% of the 3.7 million people who injected drugs (PWID) are women yet, they represented 44% of new HIV cases among PWID (*n* = 2,651) in 2024 [4, 5]. WWID’s elevated HIV risk stems in part from difficulties they face negotiating HIV prevention tools (e.g., condoms, sterile syringes) with men [6, 7]. Compared to male counterparts, WWID are also more likely to engage in survival sex or sex work [8], experience higher rates of interpersonal violence [9, 10], anxiety, depression, and post-traumatic stress disorder [11], as well as social stigma and discrimination related to their drug use [12, 13]. At the structural level, which encompasses elements of the built and social environment, geographic clusters of HIV and other infectious diseases have been identified in urban census tracts with elevated rates of crime and policing [14–17]. These areas may overlap with those where sex work and public injection drug use also cluster, which further exacerbates risk of HIV acquisition and (re)victimization among WWID.

While WWID could benefit greatly from user-driven HIV prevention strategies that do not require negotiation with a partner, such as pre-exposure prophylaxis (PrEP), they remain largely excluded from all phases of PrEP research [2, 18]. The limited literaure on PrEP among WWID, suggests many find the concept of PrEP acceptable, but face barriers to PrEP access, including limited PrEP knowledge [19], a lack of gender-sensitive health care [20] and discrimination from medical providers [21]. The result is low utilization of preventive and reproductive health care [1, 22, 23], distrust of medical institutions and providers [24, 25], and low rates of PrEP prescribing to WWID. An analysis of pharmacy claims from a large commercially insured cohort with opioid and/or stimulant use disorder (*n* ≈ 550,000) found that fewer than 0.15% of new PrEP prescriptions were issued to individuals with evidence of injection drug use, and that males were nearly 8.5 times more likely to receive PrEP than females [26].

In the few studies of PrEP adherence focused on WWID, challenges to achieving and maintaining the dosing schedule needed for oral PrEP efficacy were identified [2, 18]. Factors contributing to non-adherence include homelessness, which makes it difficult to store or discreetly take medications, and competing survival priorities, such as securing food, shelter, and procuring drugs to avoid opioid withdrawal [27]. Meeting acute survival needs often takes precedence over remembering to take medications and affects WWID’s ability to prioritize their health [27]. Despite a demonstrated need, no evidence-based adherence interventions tailored to the specific HIV prevention needs of WWID currently exist.

Long-acting injectable cabotegravir (CAB-LA) as PrEP could address a number of challenges to oral PrEP adherence because it is discreet and requires no daily action to be effective. CAB-LA is a long-acting formulation of an integrase inhibitor that is administered intramuscularly as two monthly loading doses followed by bi-monthly injections [28]. In two randomized controlled trials, one with MSM and transgender women [29] and another with cisgender women in sub-Saharan Africa [30], CAB-LA was demonstrated to be superior in efficacy to oral PrEP potentially because adherence to injections was higher than adherence to pill-taking in both studies. For CAB-LA users who miss their target dates for injections and may have lapses in protection against HIV acquisition, oral PrEP, generally with oral TDF/FTC or TAF/FTC (given barriers to accessing oral cabotegravir and less robust data on its efficacy than other PrEP options), can be used as a bridge until additional injections are given. CAB-LA is safe and well-tolerated, aside from the potential for injection site reactions that are generally mild and rarely associated with drug discontinuation. Laboratory monitoring includes testing for HIV at the time of each injection as well as STI testing every 4 months as indicated [28].

However, there is a dearth of evidence on its uptake among PWID due in part to the slow roll-out of CAB-LA nationwide, especially among women [31, 32]. Most acceptability studies suggest that WWID would prefer CAB-LA over oral formulations [33–35], however, only one study has documented real-world uptake when WWID have access to both types of products. That paper, published by our group, confirmed hypothetical product preferences, reporting that 55 of 62 (89%) selected CAB-LA over oral PrEP after discussing both options with a provider [36].

In this paper, we describe findings from narrative analyses of 25 semi-structured qualitative interviews with WWID initiating PrEP at a low barrier harm reduction program in Philadelphia (PA, USA). Our goal was to explore product choice and compare decision-making between women choosing CAB-LA versus those choosing oral PrEP, to help tailor outreach and services best matched to the specific needs of WWID considering or initiating PrEP. In our analysis, we use an intersectional lens to disentangle how lived experience, provider-patient interactions, and product attributes influence choice, and how access to care within a lower barrier clinic, operating alongside a syringe service program (SSP), helped facilitate access to PrEP.

## Methods

### Recruitment and Setting

Our analytic sample was recruited from an ongoing randomized controlled trial (“*TIARAS”*) evaluating an intervention designed to reduce HIV acquisition risk among cisgender WWID by improving psychological adjustment and supporting PrEP adherence [37]. Women were informed about TIARAS through street outreach or in-person contact (e.g., screening women waiting to access sterile syringes) at Prevention Point Philadelphia (PPP), a large multi-service harm reduction program that offers PrEP through a low barrier clinic. Women were offered a small material incentive (e.*g.*, fingernail clippers, lip balm, sanitary wipes) to interact with staff who provided basic education about PrEP, including why it is prescribed, how to access it at PPP, and the two FDA-approved PrEP regimens (daily oral or long-acting injectable). Interested women not currently prescribed PrEP were offered a warm handoff to a medical receptionist at PPP who would verify they were covered by medical insurance or help them sign up for coverage for PrEP-related costs; participants covered medication costs through insurance or other access programs. All research activities for this study and the larger *TIARAS* study occurred at PPP.

The next steps in the intake process included rapid HIV testing, followed by individualized PrEP education and counseling with PPP navigators (counselors), and specimen collection for HIV RNA, serum creatinine levels, and sexually transmitted infection screening with a trained medical assistant. Specimens were sent to an external laboratory for processing, and results typically returned within one week. Ideally, participants completed these first steps as part of a single visit. Participants selecting oral PrEP were then scheduled to return to meet with a prescriber who reviewed their results, provided STI treatment as needed, confirmed product choice, and dispensed oral PrEP. Women selecting CAB-LA were scheduled for an additional follow-up appointment (about 1-week later) to receive their first loading dose because CAB-LA needed to be delivered to the clinic.

### Eligibility Criteria

Cisgender women who completed the PrEP process at PPP and reported injecting illicit stimulants or opioids within 6-months were eligible for TIARAS if they enrolled within 2-weeks of receiving their PrEP prescription.

### Analytic Sample

For the current study exploring PrEP choice, we recruited and interviewed TIARAS participants within 30 days of their baseline TIARAS study visit.

### Procedures

From December 2022 through February 2024, we recruited a subsample of women from TIARAS. We initially aimed to balance the sample with equal representation between PrEP choice (oral PrEP vs. CAB-LA). However, most participants in TIARAS chose CAB-LA thus, we tried to enroll all WWID selecting oral PrEP to ensure sufficient representation to gain a deeper understanding of any differences in product choice. Data collection continued until we reached thematic saturation and were no longer identifying any new patterns or themes in the data [38].

Participants completed semi-structured interviews designed to elicit information about their CAB-LA versus oral PrEP choice and related decision-making. We used follow-up probes to understand how individual contexts, past medical experiences, and product attributes may have influenced product choice. For example, we asked participants to elaborate on their decision-making by posing questions like: *“Tell me more about why you chose to start [pills versus injectable PrEP]?”*; and *“Now I’d like to talk about the conversation you had about PrEP with the doctor that you saw. Thinking back to that appointment*,* please describe what they said to you about PrEP.”* We also explored participants’ rationale for initiating PrEP and their experience accessing care at PPP, in a clinic co-located with a SSP using questions like, *“I’d like you to walk me through getting PrEP care at PPP. Pretend you were telling someone who knows nothing about PrEP. How would you explain the process to them?”*; *“Were there any roadblocks or challenges to getting on PrEP?”*

Interviewers [KM, EM] were also research assistants on the TIARAS study and had been working at PPP onsite beginning in June 2022. They were known by and had developed a good rapport with many participants prior to their interview. The interviewers received training in the qualitative research techniques used in this study from AMR and AG and had opportunities to conduct a mock interviews with feedback before beginning independent data collection. In addition, each interviewer had experience working with women who use drugs prior to joining the study team. This included roles in clinical research studies and drug treatment settings. Prior to initiating recorded interviews, researchers read WWID a script that included, *“You are under no obligation to complete the interview and we can skip any question that you don’t want to answer…In this interview*,* you are the expert– there are no right answers.”* The goal of the script was to reduce researcher and participant bias by establishing an environment of openness and respect by reminding participants they could withhold information while also encouraging them to share openly when appropriate.

Face-to-face interviews took place in a private office at PPP. They were audio-recorded and lasted up to one hour. Participants received $30 cash for their time. To describe the sample, we also extracted PrEP prescription data from electronic medical records, as well as demographic, behavioral, and health care utilization data from the TIARAS baseline survey. The study was approved by the Drexel University Institutional Review Board (IRB), the Philadelphia Department of Public Health IRB, and reviewed by PPP’s research committee.

### Analysis

We used elements of “rapid qualitative assessment” to review and summarize the data [39]. This included a collaborative process of organizing transcript summaries, based on interview guide domains, into matrices while interviews were still underway. We combined this with an inductive narrative analysis in which new themes were noted and discussed as they emerged, so that authors could identify thematic saturation in real-time. Our analytic process included seven steps, initiated immediately post-interview: (1) the interviewer completed a structured debriefing form [40] that included prompts for reflective notes on a priori domains from the interview guide and emerging topics; (2) a second member of the research team completed verbatim transcription of the interview; (3) the transcriptionist completed the same debriefing form as the interviewer with an additional area to add supporting data (quotes); (4) the entire research team engaged in a weekly group discussion to review participant-level findings, guided by the interviewer and/or transcriptionist (group disagreements were resolved using consensus building); (5) a research team member synthesized findings into a structured matrix (Microsoft Excel, 2024) that included a summary of participant-level findings (notes and quotes); (6) data from the matrix was analyzed for themes across participants and for differences by group (e.g., daily oral v. CAB-LA; prior PrEP experience v. PrEP naïve); and (7) major themes and supporting evidence were discussed as a group, findings were synthesized for inclusion in the manuscript, and participant ID numbers were replaced with self-selected pseudonyms. Data from baseline surveys and electronic medical records were analyzed using Microsoft Excel 2024 and SAS Version 9.4 (SAS Institute Inc., Cary, NC, USA).

## Results

### Sample Characteristics

This racially diverse sample (*n* = 25) was primarily middle-aged (43 years) with 48% identifying as Black, Mixed Race, or Latinx (Table 1). Most women chose CAB-LA (*n* = 17, 68%). The average number of days between receiving a prescription and being interviewed was 11.5 days (IQR: 5.5–20.5). Many women reported significant structural vulnerabilities like poverty (median annual income: $3,720) and housing instability, with 16 reporting homelessness (64%). High levels of substance use were prevalent; 10 (40%) women reported daily polysubstance use over the past 3 months, and 18 (72%) reported daily injection drug use in the past month. Over two-thirds of the sample (*n* = 17, 68%) had experienced a nonfatal overdose. Eight (32%) participants reported receptive syringe sharing, and 9 (36%) reported paraphernalia sharing. Slightly over one third of participants (*n* = 9, 36%) reported having no sexual partners in the past six months. Among the women reporting sex, the average number of sexual partners was 2.5 (IQR 1-9.5). Inconsistent condom use in the past month and transactional sex in the past 6-months were reported by 7 (28%) and 12 (48%) women, respectively. Prior to TIARAS, 19 (80%) women had never taken PrEP, and 5 (20%) were re-starting PrEP after discontinuation. Those re-starting had previously used oral (1/5, 20%), injectable (2/5, 40%) or both types of PrEP (2/5, 40%).

**Table 1:** Sample characteristics (*N* = 25)

Variable	Total, *N* (%)
Age^a^	43 (39, 49)
Race	
Black or African American	8 (32%)
White	13 (52%)
More than one race	3 (12%)
Not reported	1 (4%)
Ethnicity	
Hispanic	2 (8%)
Non-Hispanic	23 (92%)
Sexual orientation	
Heterosexual/Straight	17 (68%)
Homosexual/Lesbian	1 (4%)
Bisexual	7 (28%)
Relationship status single	20 (80%)
Educational attainment	
Less than high school	7 (28%)
High school graduate/GED	6 (24%)
Some college or higher	12 (48%)
Annual income^a^	$3,720 ($3,000, $10,536)
Current living situation	
Housed	9 (36%)
Unhoused	16 (64%)
Substance Use	
Daily injection drug use (past month)	18 (72%)
Average daily injections^a^	5 (3,8)
Receptive syringe sharing (past month)	8 (32%)
Paraphernalia sharing (past month)	9 (36%)
Daily polydrug use (past 3-months)	10 (40%)
Overdose experience history	17 (68%)
Sexual Behavior	
Any sexual partner (past month)	16 (64%)
Median sex partners (past month)^a^ (*n* = 16)	2.5 (1, 9.5)
Inconsistent condom use (past month)	7 (28%)
Sex exchange (past 6-months)	12 (48%)
Bacterial STI at most recent test (past year)	8 (32%)
Health Status & Health Care Utilization	
MOUD use (past 6-months)	8 (32%)
Prior PrEP use (*n* = 24)	5 (20%)
Prior PrEP modalities used (*n* = 5)	
Oral PrEP	1 (20%)
Injectable PrEP	2 (40%)
Both	2 (40%)
PrEP modality selected at baseline	
Oral PrEP	7 (28%)
Injectable PrEP	18 (72%)

^a^Median (IQR (Interquartile Range))

### Qualitative Findings

Three major themes emerged from the in-depth interviews: (1) PrEP provides women with a valued safety net and initiation is a form of self-preservation; (2) Co-located care, small incentives, and provider respect for women’s medical autonomy sustained engagement during the multi-visit initiation process; and (3) Longer lasting HIV protection with less frequent dosing is preferrable. Each theme is detailed below.

1. PrEP provides women with a valued safety net and initiation is a form of self-preservation.

Women in this sample valued PrEP as a tool to gain/regain control over their health and reduce their worry about HIV exposure, recognizing they had engaged in activities that increased their likelihood for HIV acquisition, especially when trying to avoid painful withdrawal symptoms (i.e., sex work, reusing paraphernalia and injection equipment).

*“When you’re like in the cycle of drug addiction*,* you’re using needles*,* you kind of get in that like ‘I’m so sick*,* I don’t even care. Give me your cooker. Let me hit your cottons’ and*,* you know*,* I’m just like trying to make money so you start tricking*,* and just like the unsafe habits that you kind of forget about [HIV] when you’re in that mindset of like*,* ‘oh my God*,* I just need to get well*,*’”* (*Bomb*,* age 34*,* Non-Hispanic White*,* PrEP experienced*,* chose CAB-LA*).

However, women rarely accepted a warm hand off the first time they engaged with outreach staff. Rather, most women experienced a prolonged period of engagement, often engaging with outreach staff multiple times before seeking services. *“I was interested after I slowed down and thought about [PrEP]*,* you know? That I would rather have something that prevents it…” (T*,* age 41*,* Non-Hispanic White*,* PrEP naïve*,* chose CAB-LA).* A noted barrier to accepting a warm hand off to care was the severity of drug addiction and women’s need to prioritize more acute survival needs. Ultimately, women sought care when it was feasible for them and only after they had time to weigh PrEP benefits against their perceived likelihood for exposure and the severity of HIV disease. Several women referenced knowing people who were living with HIV or who had died from its complications.

*“I seen them deteriorate under AIDS*,* like it’s an ugly thing*,* you know*,* and that scares me a lot.… I’m glad that now I have the information and what I need to avoid it…Me knowing that I can save my own life*,* there’s nothing better than that*,*” (Angry Bone*,* age 53*,* Hispanic*,* PrEP naïve*,* chose CAB-LA)*.

In this way, PrEP initiation was seen an act of self-preservation during times of extreme vulnerability when women needed a safety net. Initiation was a way for women to exercise agency over their health during a period of their lives marked by extreme vulnerability and little control.

*“We take action on our own lives that’s*,* I think it’s good*,* you know*,* because you know drugs and alcohol take a whole lot from our lives*,* and you know*,* this is one thing… that we have control over*,* you know,” (TJ*,* age 49*,* Non-Hispanic White*,* PrEP naïve*,* chose Oral PrEP).*

(2) Co-located care, small incentives, and provider respect for women’s medical autonomy helped women navigate a multi-visit PrEP intake process.

Being able to access PrEP co-located with other harm reduction services at PPP was critically important. Prior to the study, women were already using many of PPP’s services including drop-in services where they could access clothing, food, or coffee in addition to formal programming like the syringe exchange, emergency shelter, or medical services (e.g., wound care or opioid substitution therapy).

*“I’m plugged into Prevention Point*,* so I would say Prevention Point was kind of the medium because I was in there with having a case manager and taking advantage of the other services like shelter and um the different clothing and food and all of that*,* needle exchange… they kind of was the gateway,” (LaLa*,* age 46*,* Non-Hispanic Black*,* PrEP naïve*,* chose Oral PrEP).*

Most participants had a pre-existing and supportive relationship with a staff member, such as a case manager, with whom they regularly interacted and trusted. Some women expressed that PPP staff had supported them when they had nowhere else to turn and never turned their back to them when they needed support.

*“I’m used to Prevention Point. Prevention Point is the one place I feel comfortable. I’ve gone through everything. Everything! It’s like no matter what they’re still there for me so it’s something that I value a lot. They don’t ever come out and tell me ‘no you gotta go,’” (Sally*,* age 41*,* Hispanic Black*,* PrEP experienced*,* chose CAB-LA).*

PrEP navigators and clinical staff were respected by WWID for being knowledgeable, highly skilled, and willing to take time to provide thorough care. One woman noted how much she appreciated it when a PrEP navigator took time to seek out information to answer her question, rather than brushing her off or giving a partial or inaccurate response.

*“[The PrEP Navigator] was great with whatever answers I needed*,* and if they didn’t know*,* they truly would walk off and go find the information*,*” (Angry Bone*,* age 53*,* Hispanic*,* PrEP naïve*,* chose CAB-LA).*

Many women expressed gratitude for the experienced phlebotomist who could successfully draw their blood, even when they considered themselves a *“hard stick”* due to collapsed veins or dehydration - common conditions that make specimen collection difficult in this group.

*“A lot of the obstacles for me is blood work. It’s hard for me to get anything done because I’m such a hard stick…” (Bomb*,* age 34*,* Non-Hispanic White*,* PrEP experienced*,* chose CAB-LA).*

In sharp contrast to their experiences with other medical providers, participants felt cared for by members of the PrEP team. They noted that the PrEP team honored their medical autonomy and ability to make a rational decision about the most appropriate PrEP product for their needs and circumstances. They described how product choice resulted from shared decision making, after engaging with the provider in a conversation about the benefits and disadvantages of each product in the context of their personal circumstance.

*“At any point I could actually feel free and feel comfortable to let them know if I’m not comfortable with anything or if I want to change anything,” (Lala*,* age 46*,* Non-Hispanic Black*,* PrEP naïve*,* chose Oral PrEP).*

Moreover, once their choice was made, women felt their decision was respected by providers. *“Nobody will tell you ‘you should do this over that’*,* it’s always that person’s choice. Whatever is convenient. Whatever works best. Whatever that person thinks. They just educate you on both and you make the choice,” (Sherry*,* age 43*,* Non-Hispanic Black*,* PrEP naïve*,* chose CAB-LA).* These positive interactions counterbalanced negative, stigmatizing experiences within other medical institutions, and helped alleviate frustrations when women encountered delays in moving through the PrEP initiation process.

For many women, the initiation process was burdensome; taking an average of 3 visits (IQR: 3–4) over 18 days (IQR: 10.5–28) to complete, with several participants experiencing prolonged delays in care. On average, WWID initiating CAB-LA took longer to complete the intake process than those starting oral PrEP (20 days v. 12 days, respectively). Participants noted that long wait times in PPP’s medical reception were common, in part because many accessed walk-in services and came to clinic without scheduled appointments. On days PPP was understaffed, or women arrived in the afternoon, long lines often formed, resulting in WWID waiting several hours to be seen and occasionally some were asked to return the following day though they had already waited several hours to be seen. This understandably caused frustration on the part of WWID.

*“Conflicting schedules or scheduling issues was probably one of the things that was prohibiting me. Specifically*,* there’s different individuals that obviously you have to meet with. When I would come*,* they weren’t here. They weren’t at work. They weren’t available*,* or they were here*,* and it was a full schedule*,* or their schedule cut off earlier (than it was supposed to)*,* they left early*,* or those things were happening*,*” (Sherry*,* age 43*,* Non-Hispanic Black*,* PrEP naïve*,* chose CAB-LA).*

This was challenging because *“sometimes you just get lazy*,* like it’s hard to like want to come down here or get here early rather than wait all day for that 5-minute visit or whatever,” (Bomb*,* age 34*,* Non-Hispanic White*,* PrEP experienced*,* chose CAB-LA).* However, high touch engagement with TIARAS staff (e.g., accompanying women to check-in, providing snacks while they waited), respectful treatment by PPP staff along with the provision of small incentives at important milestones (e.g., HIV testing [$5 gift card] and adherence counseling when medications are dispensed [$50 gift card]), played a crucial role in helping women stay engaged through the multi-visit initiation process. With regard to the importance of financial incentives, women described how these resources allowed them to address their survival needs without engaging in potentially health-harming behavior.

*“When you’re dealing with people in low income community that have financial difficulties*,* incentives definitely make a difference because it takes off the pressure of having to do either strenuous things for money*,* illegal things*,* or things that are hard… that [incentives] makes a heck of a difference… it is a financial struggle to survive*,* to have a roof over your head*,* to have food*,* shelter*,*”* (Sherry, age 43, Non-Hispanic Black, PrEP naïve, chose CAB-LA).

(3) Longer lasting HIV protection with less frequent dosing is preferrable.

When evaluating products, WWID carefully considered their attributes including route of administration, potential side effects, duration of effects, and dosing schedule. They weighed these attributes against their current circumstances and prior experience with oral and injectable medications. Given the instability in their lives and their urgent need to meet acute daily survival needs, including managing their drug use, WWID noted that it would be difficult to prioritize taking a daily, oral medication even when they recognized it had long term health benefits. Those selecting CAB-LA also endorsed the importance of a less demanding dosing schedule, especially if they had struggled to adhere to a daily regimen in the past. The less frequent dosing schedule was described as an easy choice.

*“… I think that you know in this like life*,* it’s so chaotic. You don’t stop and think like*,* oh I need to do something that will benefit me in the long run. So*,* just having the injection and kind of like forgetting about [it is important to me],” (Bomb*,* age 34*,* Non-Hispanic White*,* PrEP experienced*,* chose CAB-LA).*

*“It was like*,* ‘do you wanna take the pill every day or do you want to take the shot?’ I already had problems with being med-compliant*,* so it’s a no-brainer that I should choose the shot*,*” (Sara*,* age 42*,* Non-Hispanic White*,* PrEP naïve*,* chose CAB-LA).*

Concerns about storing oral medications were common, with many WWID reporting experiences of medication theft, which reinforced less frequent clinic-based dosing as a valued product attribute. *“I’ve had so many of them stolen from me*,* so many prescriptions*,* they don’t even know what they are*,* and they still take them… I like that I only have to do it once a month*,* you know*, ***the simplest way to go***,*” (T*,* age 41*,* Non-Hispanic White*,* PrEP naïve*,* chose CAB-LA).*

Finally, WWID were also concerned about *“messing up”* an oral regimen and recognized that failing to maintain the recommended dosing schedule would undermine the valued safety net that PrEP could provide. In this way, CAB-LA was considered a *“safer”* choice, and was especially valued for providing longer periods of HIV protection. One participant shared: “*I feel more safe [with the shot]. If I took the pills*,* I think I’d messed up and I believe I’ll get HIV*,*” (Luna*,* age 59*,* Non-Hispanic Black*,* PrEP naïve*,* chose CAB-LA).*

WWID who chose oral PrEP had considerably different reasons for their choice compared to those selecting CAB-LA. WWID in this group preferred a short-acting medication that could be rapidly reversed if they experienced unwanted side effects.

*“My thought is like the pill like I could take every day and if I would start to get sick like I could just stop taking it. With the injection*,* it’s in my body and I can’t get it out*,*” (Angela*,* age 31*,* Mixed Race*,* PrEP naïve*,* chose Oral PrEP).*

In a few cases, distrust of *“big pharma”* underpinned the perceived benefits of rapid reversibility as a key product attribute, with one participant noting that *“I’m not the biggest fan of pharmaceuticals and things like that*,* ‘cause you know*,* we just– a lot of times*,* we really are just like doing things in blind faith*,*” (LaLa*,* age 46*,* Non-Hispanic Black*,* PrEP naïve*,* chose Oral PrEP).* Finally, a few WWID expressed an aversion to needles.

## Discussion

This qualitative study is one of the first to examine how product attributes influence PrEP product choice among WWID with access to both oral and long-acting injectable modalities. Similar to studies assessing hypothetical choice and willingness to use PrEP [35], our data suggest WWID consider PrEP an important HIV prevention tool and have a strong preference for CAB-LA [41]. The two most salient product attributes for WWID were duration of protection and frequency of dosing. Women who chose CAB-LA preferred a long-acting medication and the possibility of achieving longer-lasting HIV protection with less day-to-day effort compared to oral PrEP. Most participants selecting oral PrEP did so because it was short-acting and placed greater value on the ability to quickly reverse any unwanted side effects, and others disliked injections. In this way, participants chose the formulation that was perceived as least risky and most beneficial for them.

Though CAB-LA is a newer product than oral PrEP, few women expressed concerns about its efficacy or side effects. When considering which product to choose, women first evaluated fit within their current circumstances, considering the impact of addiction on their ability to adhere, or housing status on their ability to safely store medications. These same barriers impacted when and how women sought care, with some needing time to process information and address daily survival needs prior to initiating the PrEP process. To encourage women to adopt PrEP, multiple client engagements may be needed. Reminders about when and how to access PrEP care and its benefits allow women to reflect and process the information on their own time. Moreover, women in this sample noted that it would be difficult to establish a healthcare routine involving a daily oral medication, which is consistent with the current literature [27, 42]. This concern was particularly salient for women who feared their oral medications would be lost or stolen as had happened to them in the past. Thus, a notable benefit of injectable PrEP is that it is administered in a clinical setting where staff are responsible for ordering and storing medications prior to use [43].

Medical distrust was common in the sample and stemmed from negative and stigmatizing interactions with health care providers operating outside of the SSP. Because PrEP services were co-located within a highly utilized and trusted harm reduction agency, women had few concerns about, and none experienced, provider stigmatization. On the contrary, welcoming staff, high-quality phlebotomists, and clinicians familiar with the healthcare needs of WWID were instrumental to overcoming distrust. Throughout the intake process, most women felt their medical autonomy was respected, which was in sharp contrast to prior experiences. Through educational sessions with the providers, women felt able to make an informed choice that would be respected by providers. These relationships were vital to keep WWID engaged throughout the PrEP initiation process which could sometimes take several weeks.

Results also support the call to expand PrEP services delivery within SSPs [44, 45]. However, few programs offer this clinical service [46]. Thus, interventions are urgently needed to reduce provider bias towards PWID in traditional health care settings [21], and to expand the of reach of prescribers skilled in patient-centered care for WWID, for example, through telemedicine [47]. PrEP implementation strategies that incorporate peer health workers have also been shown to address distrust and help patients navigate care, and could be of benefit for WWID seeking services within SSPs that offer HIV testing with referrals to outside clinical services for PrEP [48].

While the data provided by this sample are novel, they also have several key limitations. The sample is comprised of women who initiated PrEP, which is required for enrolling in the parent trial [37]. WWID’s interest could be lower or higher if PrEP was offered as a standalone clinical service, not involved with a longitudinal research project. It is also possible that the viewpoints and experiences of women who started the intake process but disengaged at mid-point may be different from women in this study, which has a focus on early stages of the PrEP continuum. We do not yet have data on adherence or persistence to PrEP modalities for WWID, though the robust contraceptive literature demonstrates that when women have access to a broader range of widely varying products, initiation increases [49], especially when coupled with individually-tailored counseling [50], a key tenet of patient-centered care and harm reduction programs. The study is also set in Philadelphia (PA, USA), where there was recently an HIV outbreak among people who use injection drugs [51]. PrEP may have lower perceived benefits to WWID living in settings with lower HIV incidence. It is also the case that many SSPs are unable to provide the same level of robust clinical services as PPP [46]. In settings where PrEP services are not embedded and referrals to outside providers are the standard of care, women are likely to face an entirely new set of barriers to accessing PrEP [52].

### Public Health Implications

This study demonstrates the value of offering women a choice of PrEP products, the importance of supporting patient autonomy, and the benefits of SSP-based PrEP. It also suggests that duration of protection for PrEP is a major driver of product preference for WWID. This is a key finding given the prospect of even longer-acting medications like twice-yearly lenacapavir, which was recently demonstrated to have 100% efficacy in preventing HIV acquisition among cisgender women in Africa [53] and is expected to be approved for prescribing in the U.S. in June 2025. While this study fills gaps in the literature about PrEP product decision-making, it also raises new questions relating to PrEP persistence among women on CAB-LA, including, whether WWID’s perceptions of PrEP products evolve after periods of use, how well they adhere to dosing schedules, how providers evaluate the risks and benefits of prescribing longer-acting PrEP for WWID, and the multi-level factors supporting or challenging CAB-LA implementation within an SSP. Over time, our group hopes to address each of these novel areas.
